# Transgenic line for characterizing GABA-receptor expression to study the neural basis of olfaction in the yellow-fever mosquito

**DOI:** 10.3389/fphys.2024.1381164

**Published:** 2024-03-28

**Authors:** Angela Rouyar, Anandrao A. Patil, Melissa Leon-Noreña, Ming Li, Iliano V. Coutinho-Abreu, Omar S. Akbari, Jeff A. Riffell

**Affiliations:** ^1^ Department of Biology, University of Washington, Seattle, WA, United States; ^2^ Division of Biological Sciences, Section of Cell and Developmental Biology, University of California, San Diego, La Jolla, CA, United States

**Keywords:** GABA, mosquito, olfaction, GABA receptor, transgenic

## Abstract

The mosquito *Aedes aegypti* is an important vector of diseases including dengue, Zika, chikungunya, and yellow fever. Olfaction is a critical modality for mosquitoes enabling them to locate hosts, sources of nectar, and sites for oviposition. GABA is an essential neurotransmitter in olfactory processing in the insect brain, including the primary olfactory center, the antennal lobe. Previous work with *Ae. aegypti* has suggested that antennal lobe inhibition via GABA may be involved in the processing of odors. However, little is known about GABA receptor expression in the mosquito brain, or how they may be involved in odor attraction. In this context, generating mutants that target the mosquito’s olfactory responses, and particularly the GABAergic system, is essential to achieve a better understanding of these diverse processes and olfactory coding in these disease vectors. Here we demonstrate the potential of a transgenic line using the QF2 transcription factor, GABA-B1^QF2−ECFP^, as a new neurogenetic tool to investigate the neural basis of olfaction in *Ae. aegypti.* Our results show that the gene insertion has a moderate impact on mosquito fitness. Moreover, the line presented here was crossed with a QUAS reporter line expressing the green fluorescent protein and used to determine the location of the metabotropic GABA-B1 receptor expression. We find high receptor expression in the antennal lobes, especially the cell bodies surrounding the antennal lobes. In the mushroom bodies, receptor expression was high in the Kenyon cells, but had low expression in the mushroom body lobes. Behavioral experiments testing the fruit odor attractants showed that the mutants lost their behavioral attraction. Together, these results show that the GABA-B1^QF2−ECFP^ line provides a new tool to characterize GABAergic systems in the mosquito nervous system.

## 1 Introduction

Neurotransmitters play essential roles in the insect motor and central nervous systems. Gamma-aminobutyric acid (GABA), an inhibitory neurotransmitter, is one of the most highly expressed neurotransmitters in the central nervous system of insects (Homberg, 2002; Rogers et al., 2004; Enell et al., 2007). It is highly expressed in many brain regions, including the primary olfactory center, the antennal lobe (AL) (Okada et al., 2009), the optic lobe (Raghu et al., 2013), the mushroom bodies (MBs) (Yasuyama et al., 2002)—a site involved in learning and memory (Heisenberg et al., 1985; Davis, 1993), and the central complex (Enell et al., 2007). Accordingly, GABA is involved in many different sensory behaviors, including the processing of odors (Waldrop et al., 1987; Stopfer et al., 1997; Olsen and Wilson, 2008) and visual (Brotz et al., 2001; Freifeld et al., 2013) and auditory stimuli (Loh et al., 2023). The neuronal circuits and behaviors associated with learning and memory are also modulated by GABA, with GABAergic processes that broadly innervate the MBs (Liu and Davis, 2009). GABA is also involved in locomotion (Leal and Neckameyer, 2002), with many of the descending neurons from the brain being GABAergic (Hsu and Bhandawat, 2016) and are important for motor control and odor navigation (Tastekin et al., 2018).

GABA receptors—the membrane-bound receptor to which GABA binds—are important research foci in neurobiology and pest control (Hosie et al., 1997). GABA receptors occur in two types: 1) GABA_A_ type receptors, members of the ionotropic ligand-gated channel family, and 2) metabotropic GABA_B_ type receptors, members of the G-protein coupled receptor family. The GABA_A_ receptor have three subunit class encoded by three genes: *Rdl* (resistance to dieldrin; (Ffrench-Constant et al., 1991)); *Grd* (GABA and glycine-like receptor of *Drosophila*; (Harvey et al., 1994)) and *Lcch3* (ligand-gated chloride channel homologue 3; (Henderson et al., 1993)), and the subunits named accordingly, RDL, GRD and LCCH3. The receptor can form a homomeric complex with the subunit RDL or a heteromeric association of RDL and LCCH3 subunits (Dupuis et al., 2010). Three GABA_B_ receptor subtypes, GABA-B1, -B2, and -B3, have also been identified in *Drosophila*. While GABA-B1 and -B2 exhibit significant sequence similarity to mammalian GABA_B_R1 and R2, respectively, the receptor GABA-B3 appears to be an insect-specific subtype. While the GABA-B3 displays a unique expression pattern, the GABA-B1 and GABA-B2 subtypes coexpressed in the similar regions in central nervous system (Mezler et al., 2001). Together these subtypes form a heterodimer resulting to a functional GABA_B_ receptor (Galvez et al., 2001; Mezler et al., 2001).

The region- and cell-specific expression of these receptors can shape neural processing. For example, in the *Drosophila* AL, the inhibitory local interneurons (LNs) do not express either of the GABA receptor types; however, the projection neurons (PNs) express both receptors (Okada et al., 2009). The combined expression in the PNs is thought to modulate the neurons at the odor onset (via GABA_A_) and inhibit the PNs at longer timescales by the metabotropic GABA_B_ receptors (Lei et al., 2002; Wilson and Laurent, 2005). Subtle region-specific differences in receptor expression can also occur. GABA_A_ and GABA_B_ receptors are highly expressed in the AL, mushroom body calyces, and regions in the optic lobe, and central complex, but may not overlap in subregions in the mushroom body calyces, which has been suggested as a spatial separation of slow and fast GABA transmission (Enell et al., 2007). Beyond cell- and region-specific expression, GABA receptors are targets of insecticides, including organochlorine, dieldrin, and fipronil insecticides that bind to the transmembrane regions and the resistant to dieldrin (Rdl) subunit to serve as receptor antagonists (Casida and Durkin, 2015; Ozoe, 2021). Antagonist binding of the GABA receptor blocks the activity of the GABA-gated chloride channels, increasing neuronal excitation.

A further example of the importance of the GABAergic system on mosquito behavior comes from the role of GABA receptors in processing olfactory information, including the odors of nectar sources and hosts. Inhibition in the antennal lobe, the primary olfactory system in the mosquito brain—mediated by GABAergic local interneurons—is critical for the enhancement of processing specific odors, including those that represent attractive odorants (Lei et al., 2002; Olsen and Wilson, 2008; Liou et al., 2018; Lahondère et al., 2020). This process can boost the signal of relatively weak, behaviorally important, odor input while suppressing the input from the background, or repellent, odors. In *Ae. aegypti*, inhibition is essential for processing odor mixtures, like those from flowers or hosts (Lahondère et al., 2020).

Despite the importance of GABA in mosquitoes, only a handful of studies have examined GABA expression in the mosquito brain (Lahondère et al., 2020; Loh et al., 2023; Singh et al., 2023), and none have examined GABA-receptor expression. In particular, the GABA-B1-receptors are involved in many vital physiological processes and constitutes a promising pharmacological target in insecticide development where resistance problems targeting GABA_A_ receptor subunit are now observed (Zulfa et al., 2022).

Here, we use the Q-system to characterize the GABA-B1-receptor expression in the mosquito brain. The Q-system is a binary expression system that has allowed the characterization of cell and circuit function in the mosquito (Riabinina et al., 2016) and consists of a transcription factor, QF, often inserted downstream of a gene of interest, which, when crossed with a QUAS reporter line, binds to the QUAS component that is upstream of genes coding for fluorescent proteins. This system was used to insert the QF2 transcription factor in the *GABA-receptor* gene locus, thereby knocking out the gene, and the resulting line was crossed to the QUAS-mCD8GFP reporter line to characterize GABA receptor expression of olfactory regions in the *Ae. aegypti* brain. Using this novel knock-in and knock-out approach, we asked the following questions: 1) How is the GABA-receptor expressed in olfactory regions of the mosquito brain, such as the AL and MB? 2) How does the knock-out of the receptor influence parameters of the mosquito life cycle?; and 3) How does the loss-of-function of the GABA-B1 receptor influence attraction to sources of nectar or fruit?

## 2 Materials and methods

### 2.1 Insect rearing

*Ae. aegypti* lines, from BEI Resources (Manassas, VA, United States) (*Ae. aegypti*: Rockefeller, Liverpool) were raised at the University of Washington campus. Mosquito lines provided were used in immunohistochemistry and behavioral experiments. The Liverpool line was used for transgenic lines as the genetic background. The mosquitoes were maintained in BSL2 insectary at 27°C, 70%–80% of relative humidity and at a photoperiod cycle of 12 h light/12 h dark. The eggs were hatched in plastic trays and in deoxygenated ultra-pure water. Larvae were maintained under the same conditions and fed with fish food (First Bites semi-buoyant granule, Hikari Tropical), and female mosquitoes were fed from heparinized bovine blood (Lampire Biologicals Laboratory Inc., Pipersville, PA 18947).

### 2.2 Genetic construction

#### 2.2.1 QF2 driver plasmid construction

**FIGURE 1:**
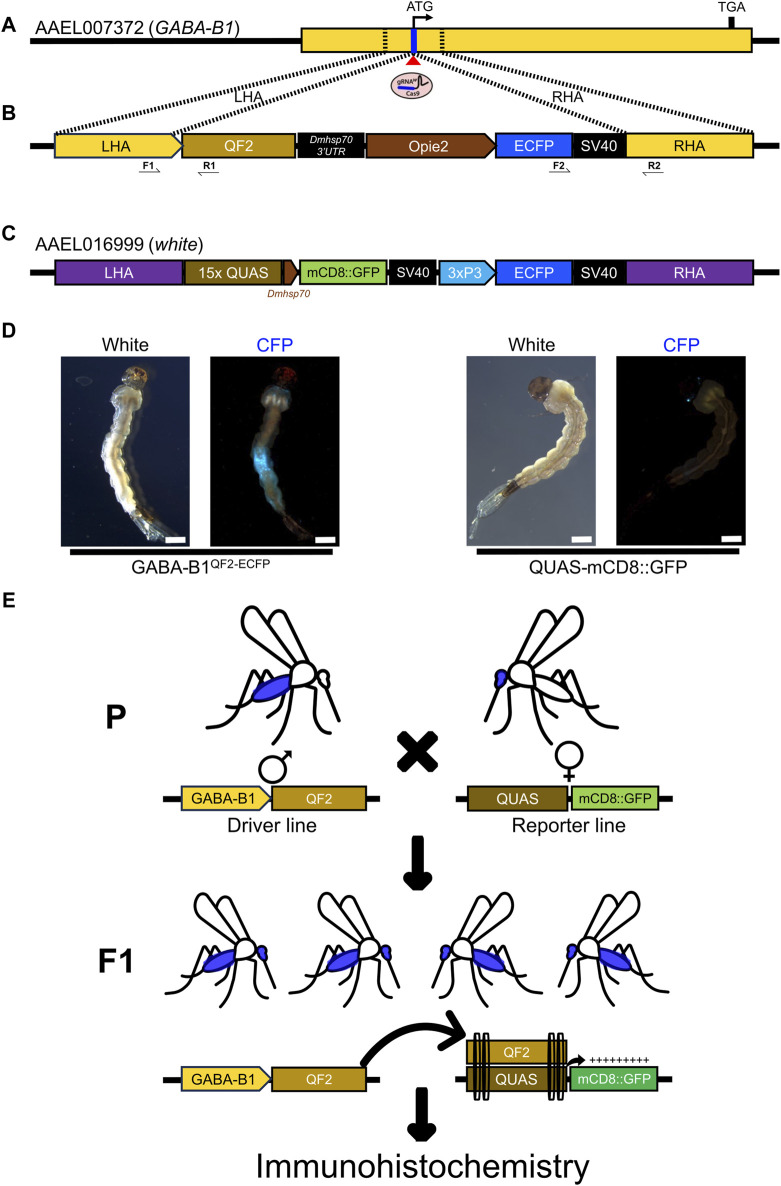
Generating GABA-B1^QF2−ECFP^ driver line. **(A)** The schematic representation of a *GABA-B1* gene structure and target gRNA location around the translational start codon (ATG). The Blue line represents the target gRNA location and the red triangle indicates a predicted Cas9 cleavage site. The dotted black lines represent the left and right homology arms from the genomic locus region selected to make a knock-in plasmid construct. **(B)** The schematic representation of the *GABA-B1* gene targeting homology-directed repair (HDR) knock-in construct flanked with left and right homology arms, the QF2 transcription factor expression cassette, and the Enhanced Cyan Fluorescent Protein (ECFP) screening marker expression cassette. Black arrow indicates genotyping primers to determine the left and right-side integration sites of knock-in. **(C)** Schematic representation of the sex-linked *white* gene targeting QUAS line components; *mCD8::GFP* reporter gene expression cassette and *3xP3* promoter driving ECFP screening marker. **(D)** The transgenic GABA-B1^QF2−ECFP^ and QUAS-mCD8GFP larvae showing presence of ECFP screening markers in the abdomen and eyes, respectively (Scale bar = 2 mm). The larvae were examined under the fluorescence microscope using cyan fluorescence protein (CFP) filter and white light. **(E)** Schematic representation of the QF2/QUAS binary expression system crossing scheme. The homozygous parents (♂) of driver line expressing QF2 transcription factor under the *GABA-B1* gene promoter crossed with the homozygous parents (♀) of QUAS reporter line. The F_1_ progeny obtained from this crossing scheme will have both driver and reporter components. In the F_1_ progeny, QF2 binds to QUAS enhancer to induce the expression of downstream reporter gene *mCD8::GFP* in *GABA-B1* gene-expressing cells or tissues, which was further visualized by immunohistochemistry.

In this research, we studied the role of G-protein coupled receptors (GPCR) family gene: *GABA-B1*. We have applied a CRISPR/Cas9 mediated homology-directed repair (HDR) knock-in approach to knockout *GABA-B1* gene and use its regulatory sequences to drive the expression of QF2 transcription factor. To make a HDR knock-in construct, first we retrieved the *Ae. aegypti* (LVP_AGWG strain) *GABA-B1* gene and transcript sequence from VectorBase [VectorBase (2022); *GABA-B1* (GPRGBB1: AAEL007372)]. Then, gene and transcript sequences were aligned to find the target gRNA sequence around the translational start codon using online tool CHOPCHOP [CHOPCHOP (2024); AAEL007372-gRNA: TAA​CCA​CGG​TAG​ATC​TTC​AT]. Based on the predicted Cas9 cleavage site for the gRNA, homology arms were selected for knock-in plasmid construction. The nucleotide sequences (∼1 kb) upstream of translation start codon (22 bp upstream of Cas9 cleavage site) was used as a left homology arm and the nucleotide sequences (−1 kb) downstream of Cas9 cleavage site was used as a right homology arm to build a knock-in construct (Figures 1A,B). The homology arm fragments, QF2 expression fragment (*QF2-Dmhsp70-3′UTR*) and Opie2 promoter driving Enhanced Cyan Fluorescent Protein (ECFP) screening marker expression fragment (*Opie2-ECFP-SV40*), were synthesized commercially (GenScript) and assembled using Gibson enzymatic assembly (Gibson et al., 2009). Then, DNA assembly reaction mixture transformed into chemically competent *E. coli* cells (Zymo Research, JM109 Cat #T3005) and the plasmids were isolated from positive colonies (Zymo Research, Zyppy plasmid miniprep kit, Cat. #D4036) and their nucleotide sequences were confirmed thoroughly using Oxford Nanopore Sequencing at Primordium Labs (Primordium Labs, 2023). For microinjection, plasmid was maxi-prepped (Zymo Research, ZymoPURE II Plasmid Maxiprep kit, Cat. #D4202).

#### 2.2.2 Generating GABA-B1^QF2−ECFP^ transgenic lines

The QF2 driver transgenic strain was generated by microinjecting preblastoderm stage embryos (0.5–1 h old) with a mixture of the knock-in donor plasmid (100 ng/ul), sgRNA (100 ng/μL) and Cas9 protein (100 ng/μL). The embryo collection, microinjections, transgenic lines generation, and rearing were performed following previously established procedures (Bui et al., 2020; Li et al., 2020; 2021).

#### 2.2.3 Genotyping and homozygosity of GABA-B1^QF2−ECFP line^

To establish a homozygous QF2 line, ECFP screening markers expressing larvae (G_0_) were maintained individually until they emerged as adults. To set up G_0_ crosses, a single ECFP marker expressing virgin female or male was crossed with a wild type (WT) single virgin female or male, and then females were blood-fed after 3 days of crossing and allowed to lay eggs on the wet paper (Figure 1E). Then, G_0_ parents were collected for genomic DNA isolation to determine the insertion sites. To recover transgenic mosquitoes, G_1_ larvae were screened under the Leica M165FC fluorescent stereomicroscope. Fluorescence was visualized using the CFP/YFP/mCherry triple filter, and the ECFP-positive larvae were selected from each cross and maintained separately. To determine the insertion sites for the knock-in construct, genomic DNA was isolated (Zymo Quick-DNA Miniprep Plus Kit Cat. No: D4068) from the G_0_ parents whose progenies were ECFP positive. For genotyping PCR, primers were designed on both sides of the knock-in site (Figure 1B) and PCR was performed using the genomic DNA template, *OneTaq* DNA polymerase (NEB, Cat. No: M0484S), and primers listed in Supplementary Table S1. Then, amplified PCR fragments were gel analyzed, purified, and the nucleotide sequences were confirmed by Sanger sequencing (Supplementary Figure S1). The genotyping PCR insertion sites determined parents’ ECFP positive progeny (G_1_) were only maintained and used for further inbreeding to establish a homozygous line (Figure 1D).

#### 2.2.4 QUAS reporter line

The QUAS-mCD8GFP reporter line (white-quas-mcd8-gfp) was generated (Craig Montell lab) by CRISPR/Cas9 mediated HDR knock-in of second coding-exon of the sex-linked *white* gene (AAEL016999), which is essential for eye pigmentation (Figure 1C). The QUAS line contains two expression cassettes; *mCD8::GFP* reporter gene expressed at basal level under the control of *D. melanogaster heat shock protein 70* (*Dmhsp70*) gene core promoter whereas the ECFP screening marker expressed in the eyes under *3xP3* promoter (Figure 1D). The QUAS enhancer copies (15x) are located upstream of the *Dmhsp70* gene core promoter, which can induce the expression of downstream *mCD8::GFP* reporter gene upon binding of QF2 transcription factor.

### 2.3 Immunohistochemistry

#### 2.3.1 Staining

For the immunostaining, GABA-B1^QF2−ECFP^ > QUAS-mCD8GFP females and males aged between 4 and 8 days old were used. Following an adapted protocol (Shankar and McMeniman, 2020), animals were anesthetized at 4°C and whole-body mosquitoes were fixed in Millonig’s buffer: 4% paraformaldehyde in 0.1 M Millonig’s Phosphate Buffer pH 7.4 (Electron Microscopy Sciences, 11582-10) with 0.25% Triton-X 100 for 3 h at 4°C. Brains were dissected in cool 0.1 M PBS pH 7.4 with fine forceps (Dumont #5, 100 nm tips) to carefully remove the head capsule as well as the pigmented ommatidia over the optic lobes and any floating air sacs connected to the brain. After washing brains 3 times for 20 min each with 0.1 M PBS pH 7.4 with 0.25% Triton-X 100 (PBST) at room temperature, brains were incubated overnight at 4°C in a blocking solution consisting of 2% normal goat serum (NGS) and 4% Triton-X 100 in 0.1 M PBS pH 7.4. Then, brains were washed 3 times for 20 min each in PBST and incubated for 3 days at 4°C in the primary antibody solution containing mouse anti-Brp (DSHB, nc82-s, AB_2314866, 1:50 v/v) targeting the pre-synaptic active zone protein Bruchpilot (Brp) (Hofbauer et al., 2009) and rabbit anti-GFP (Invitrogen, A-6455, 1:100 v/v) targeting mCD8GFP. Following another 3 washes with PBST, the brains were exposed to a secondary antibody solution for 3 days at 4°C. The secondary antibody solution consisted of goat anti-mouse Cy3 (Jackson ImmunoResearch, AB_2338680, 1:200 v/v) and goat anti-rabbit Alexa Fluor 488 (Invitrogen, A-11008, 1:10200 v/v). Finally, the brains were washed 3 times for 20 min each with PBST at room temperature, incubated in SlowFade Diamond Antifade Mountant solution (Invitrogen, S36936) overnight at 4°C and mounted between glass slides and raised coverslip to avoid tissue distortion.

**FIGURE 2:**
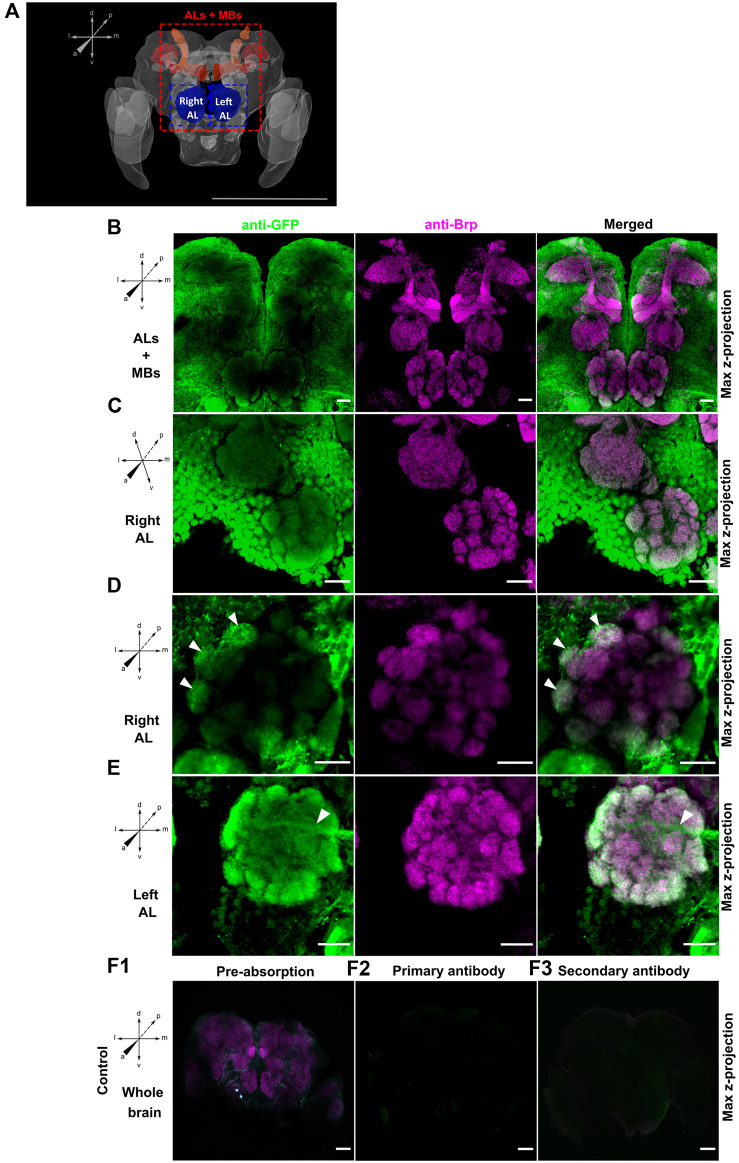
**(A)** Adapted schema of the *Ae. aegypti* brain from Insect Brain Database (Database, 2024) representing in red the mushroom bodies (MBs) and in blue the antennal lobes (ALs). Maximal projection view of confocal image stacks from male **(A,B,D and E)** and female **(C)** GABA-B1^QF2−ECFP^ > QUAS-mCD8GFP mosquito brains stained with anti-GFP antibody (green) and anti-Brp antibody (magenta). **(B)** View of antennal lobes (ALs) and mushroom bodies (MBs) area reveals that green fluorescent protein (GFP) expression is mainly present around the ALs while no expression is observed in the MBs. **(C)** Right antennal lobe (Right AL). GFP immunofluorescence shows expression of GABA-B1 in lateral and middle cell body clusters. **(D)** Right antennal lobe (Right AL). Neurons expressing the GABA-B1 receptor ramify multiple glomeruli (arrows). **(E)** Left antennal lobe (Left AL). GFP immunostaining overlaps with the presynaptic staining background in the peripheral glomeruli of ALs. A tract of GABA-B1-positive fiber entering the AL from the lateral side can be also seen (arrow). Different controls using different GABA-B1^QF2−ECFP^ > QUAS-mCD8GFP adult male brains were performed to test labeling specificity. **(F1)** The brain was preabsorbed with GFP protein. The preabsorption had almost abolished the GFP labeling without affecting the Bruchpilot (Brp) labeling. For the two other controls, either **(F2)** secondary antibodies or **(F3)** primary antibodies were omitted. Both controls showed no tissue labeling. Scale bars, 500 µm **(A)** and 20 µm **(B–F)**.

To test the specificity of this green fluorescent protein (GFP) antibody, we performed a preabsorption control where the primary antibody was preabsorbed with 1 mg/mL GFP protein overnight at 4°C before being used to incubate brains in the primary antibody solution. As the GFP protein and the Alexa Fluor 488 have the same emission spectrum and to ensure that the fluorescence observed on the brains is not due to GFP protein residues remaining despite washing, the Alexa Fluor 488 was replaced with Alexa Fluor 405. Controls where the primary antibody alone and the secondary antibody alone were also performed to confirm the specificity of the GFP antibody labeling (Figure 2F1, F2, F3).

#### 2.3.2 Immunohistochemistry image acquisition settings

Brain tissue was imaged using A1R HD25 laser scanning confocal microscope. A ×20 objective lens (0.75 NA, Plan-Apochromat) was used to capture the whole brain and AL and MB areas. Excitation of Cy3 signal was achieved with a 561 nm solid-state laser line between 0.5% and 2% laser power, a detector offset at 40 and a GaAsP PMT detector gain between 10 and 20. A 488 nm laser line was used to excite Alexa Fluor 488 with the same laser setting used for the Cy3 signal. The images were acquired with 2048 × 2048-pixel resolution.

### 2.4 Bioassays

The fitness experiments were performed using GABA-B1^QF2−ECFP^ female and male mosquitoes. Larvae were screened at stage 4 of larval development using an epifluorescence microscope to ensure that all mosquitoes had the genetic insertion.

#### 2.4.1 Wing size

**FIGURE 3:**
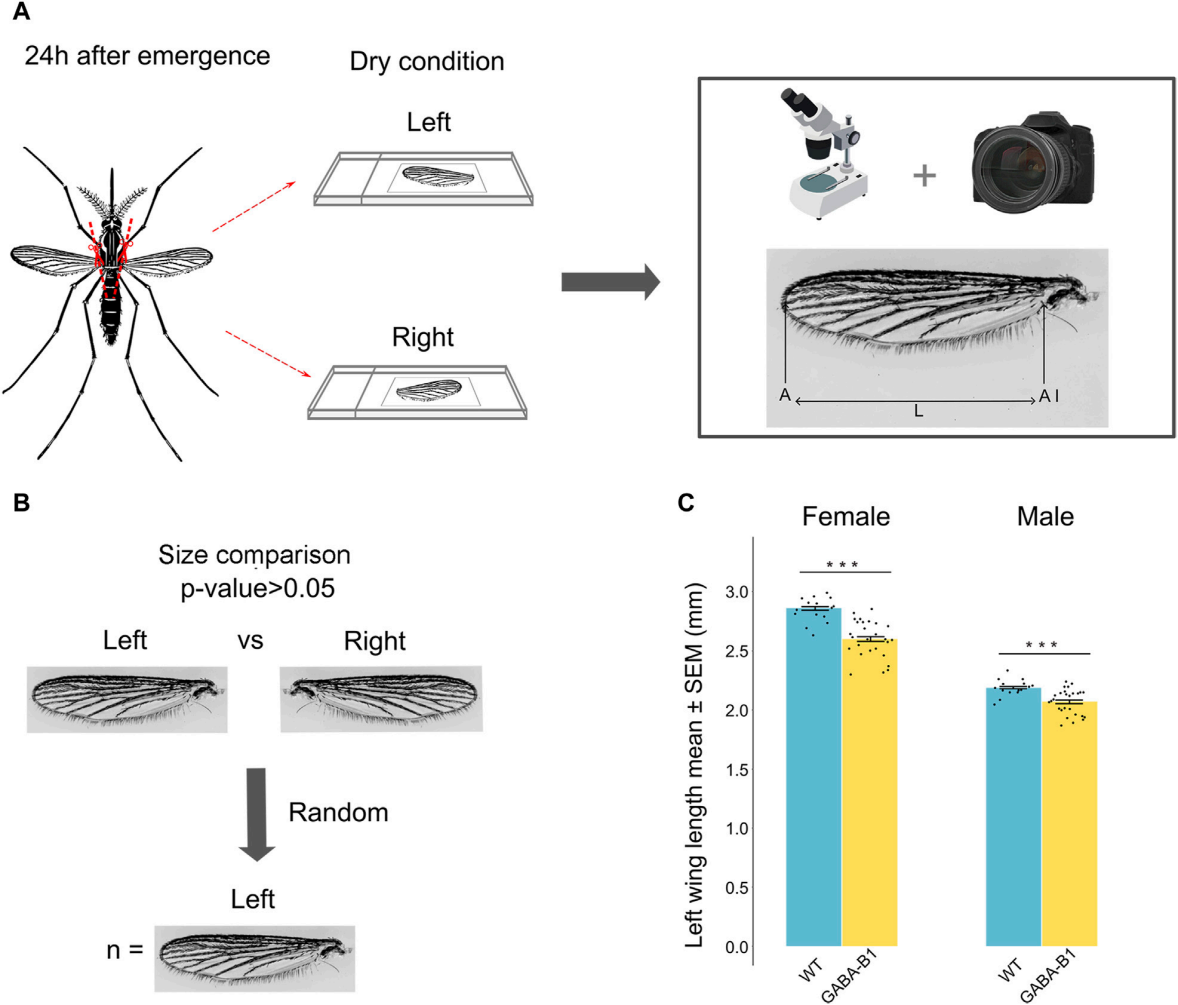
**(A)** Protocol and method used to collect the wing and to measure its size defined as the length (L) from the apex of the wing **(A)** to the axillary incision (AI), excluding the marginal fringe. Left and right wings were cut and measured. **(B)** No significant difference was observed between left and right wing (Student’s t-test and Mann-Whitney *U* test *p* > 0.05). The left wing was randomly chosen for the analysis. **(C)** Data are plotted as length mean of the left wing ±standard error of the mean (SEM) according to the sex and the line. Wild type (WT), GABA-B1^QF2−ECFP^ (GABA-B1). Each black dot represents one measure: WT female: *n* = 15, WT male: *n* = 15, GABA-B1^QF2−ECFP^ female: *n* = 27, GABA-B1^QF2−ECFP^ male: *n* = 27. Student’s t-test; *** = *p* < 0.001, ** = *p* < 0.01, * = *p* < 0.5, ns = not significant.

To prepare the wings for size measurements, 1-day-old mosquitoes were anesthetized using a cooling method. Both left and right wings of each mosquito were removed using fine forceps. Then, the wings were mounted on microscope slides under dry conditions, ensuring a flat position without any folds or distortions. To capture images of the wings, a digital camera (Nikon model D3400) mounted with an adapter on a Leica binocular microscope with a ×20 objective and adjusted to ×4 magnification was used. The length of each wing was measured directly on the captured wing images using ImageJ software. Measurements were taken from the apex of the wing to the axillary incision, excluding the marginal fringe (Packer and Corbet, 1989; Pelizza et al., 2013) (Figure 3A). To convert the wing length from pixel units to millimeters, a picture of an eyepiece graticule taken under the same conditions as the wing slides was utilized. The left wing was chosen at random in each mosquito for analysis because the differences between the length of right and left wings were not significant (Figure 3B).

#### 2.4.2 Dry weight

**FIGURE 4:**
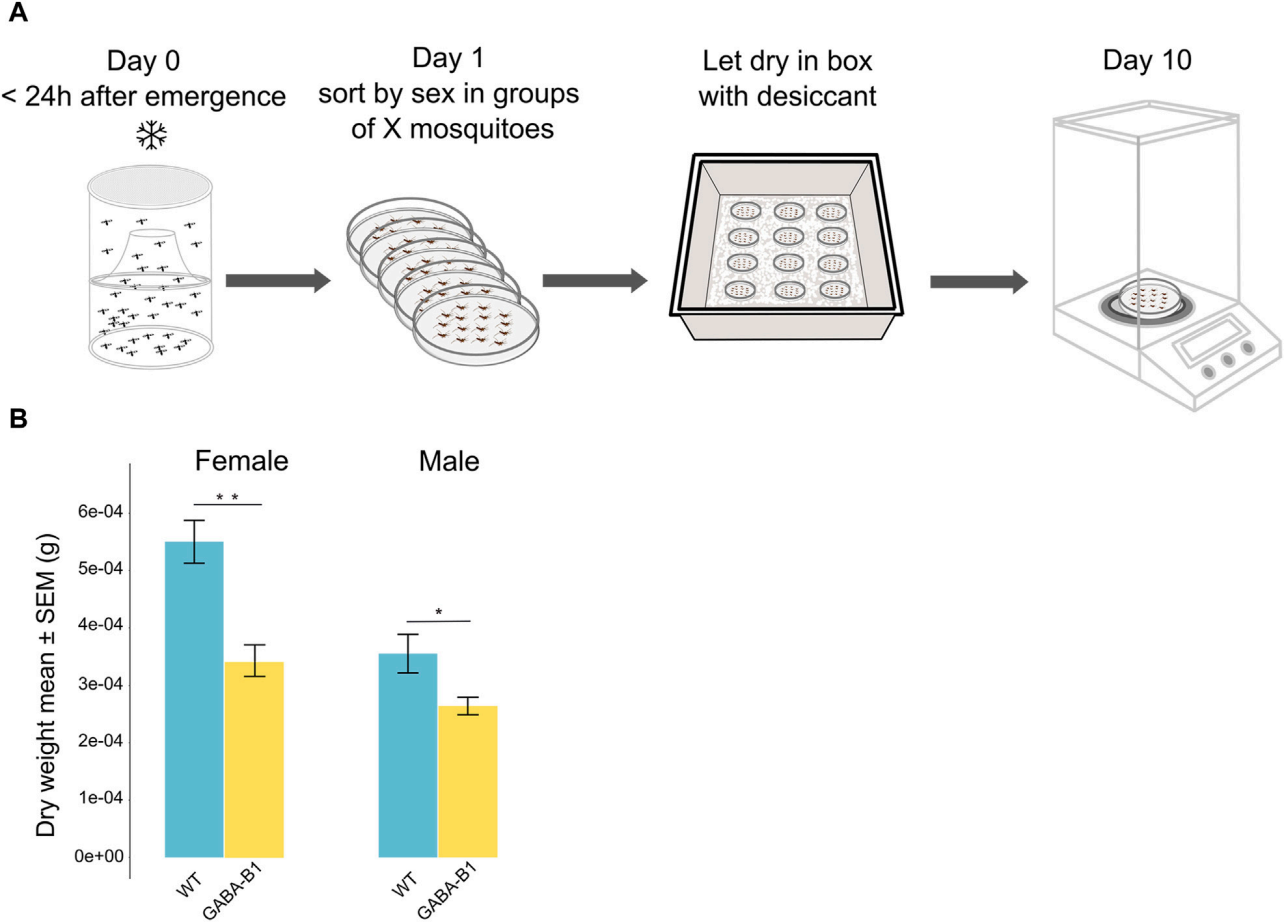
**(A)** Protocol to estimate mosquito dry weight. **(B)** Data are plotted as the mean of the dry weight ±standard error of the mean (SEM) according to the sex and the line: Wild type (WT), GABA-B1^QF2−ECFP^ (GABA-B1). The estimated dry weight of one mosquito was determined by the total dry weight of the group divided by the number of mosquitoes in each group (the number of groups: WT female *n* = 8; WT male *n* = 10; GABA-B1^QF2−ECFP^ female n = 17; GABA-B1^QF2−ECFP^ male *n* = 15). Mann-Whitney *U* test; *** = *p* < 0.001, ** = *p* < 0.01, * = *p* < 0.5, ns = not significant.

To determine the dry weight of the WT and the GABA-B1^QF2−ECFP^ lines, mosquitoes from each line were collected within 24 h of emerging and killed by placing them at 4°C for 1 day. The mosquitoes were sorted by sex and grouped within Petri dishes. The number of mosquitoes per group ranged between 12 and 37 mosquitoes. These Petri dishes were then placed inside a lidded box containing anhydrous calcium sulfate desiccant granules at room temperature. The mosquitoes remained in this condition for 10 days, allowing the desiccant to dehydrate and remove the moisture from the specimens. Once the desiccation period was complete, the dry weight of each group of mosquitoes was measured using a precise scale (Figure 4A). The estimated dry weight of one mosquito was determined by the total dry weight of the group divided by the number of mosquitoes in each group.

#### 2.4.3 Number of eggs laid

**FIGURE 5:**
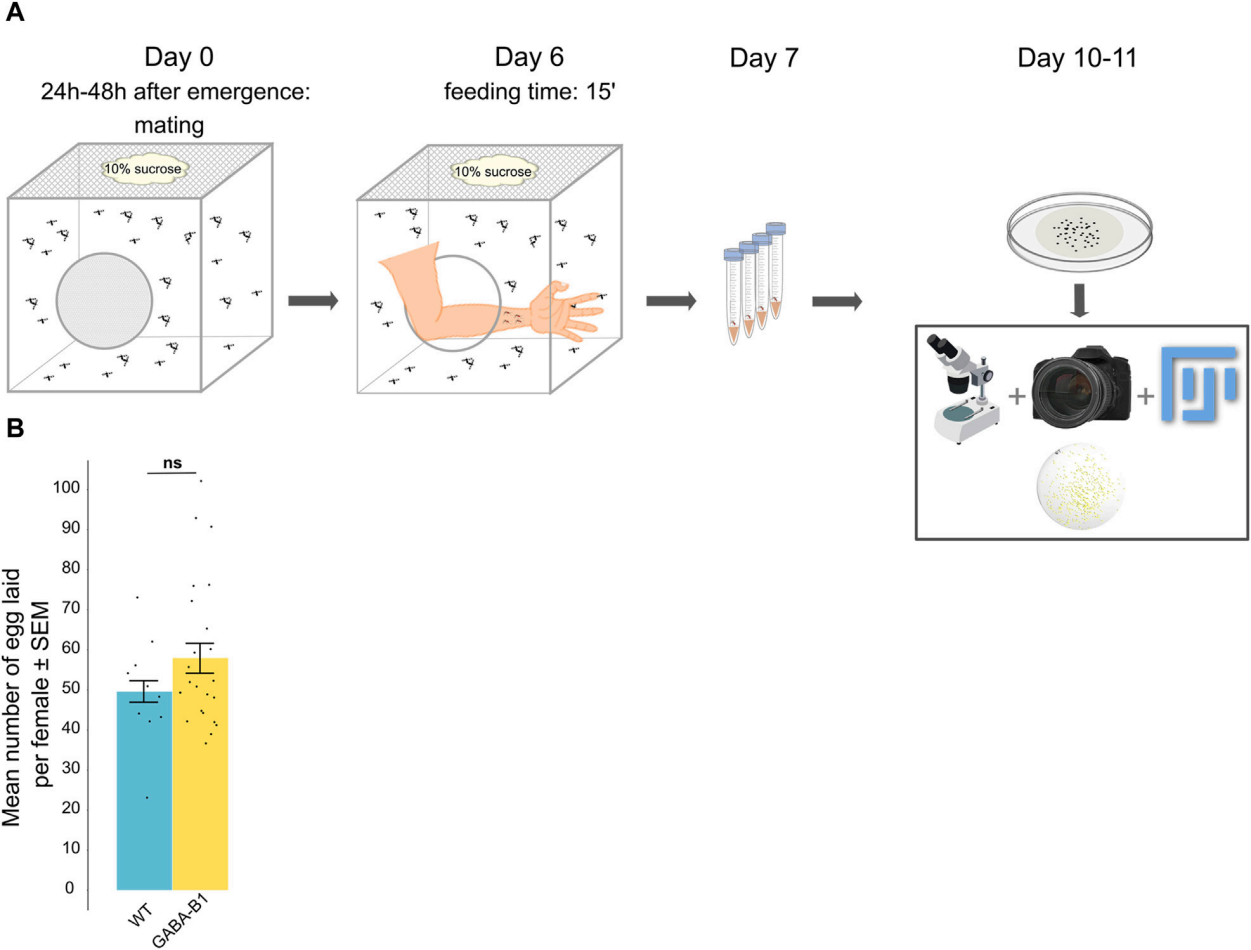
**(A)** Protocol for counting the number of eggs laid by one female. **(B)** Data are represented by the mean number of eggs laid per female ±standard error of the mean (SEM) according to the line: Wild type (WT), GABA-B1^QF2−ECFP^ (GABA-B1). Each black dot represents the number of eggs laid per female (number of females: WT n = 10; GABA-B1^QF2−ECFP^ n = 24). Mann-Whitney *U* test; *** = *p* < 0.001, ** = *p* < 0.01, * = *p* < 0.5, ns = not significant.

In order to limit the effects of various physiological processes and external factors that can impact fertility, we have chosen to compare only fecundity between the WT and GABA-B1^QF2−ECFP^ lines. For this, 1 to 2-day-old female and male mosquitoes from the same line were placed together in a rearing cage (Bugdorm-1, MegaView Science Co., Ltd. Taichung 407008, Taiwan) for a period of 6 days and were fed 10% sucrose. The sex ratio within the cage was maintained at 1 female for every 3 males. After the 6-day period, the female mosquitoes were blood-fed and the following day fed females were individually transferred to 50 mL plastic tubes containing moist filter paper placed along the inner perimeter to allow the females to lay their eggs on the moist paper. 3–4 days after the ingestion of blood, once oviposition had occurred, the filter papers with the eggs were removed. A digital camera (Nikon model D3400) was used to capture images of the flat filter papers, and the software ImageJ was utilized to count the number of eggs laid by individual females (Figure 5A).

#### 2.4.4 Longevity

**FIGURE 6:**
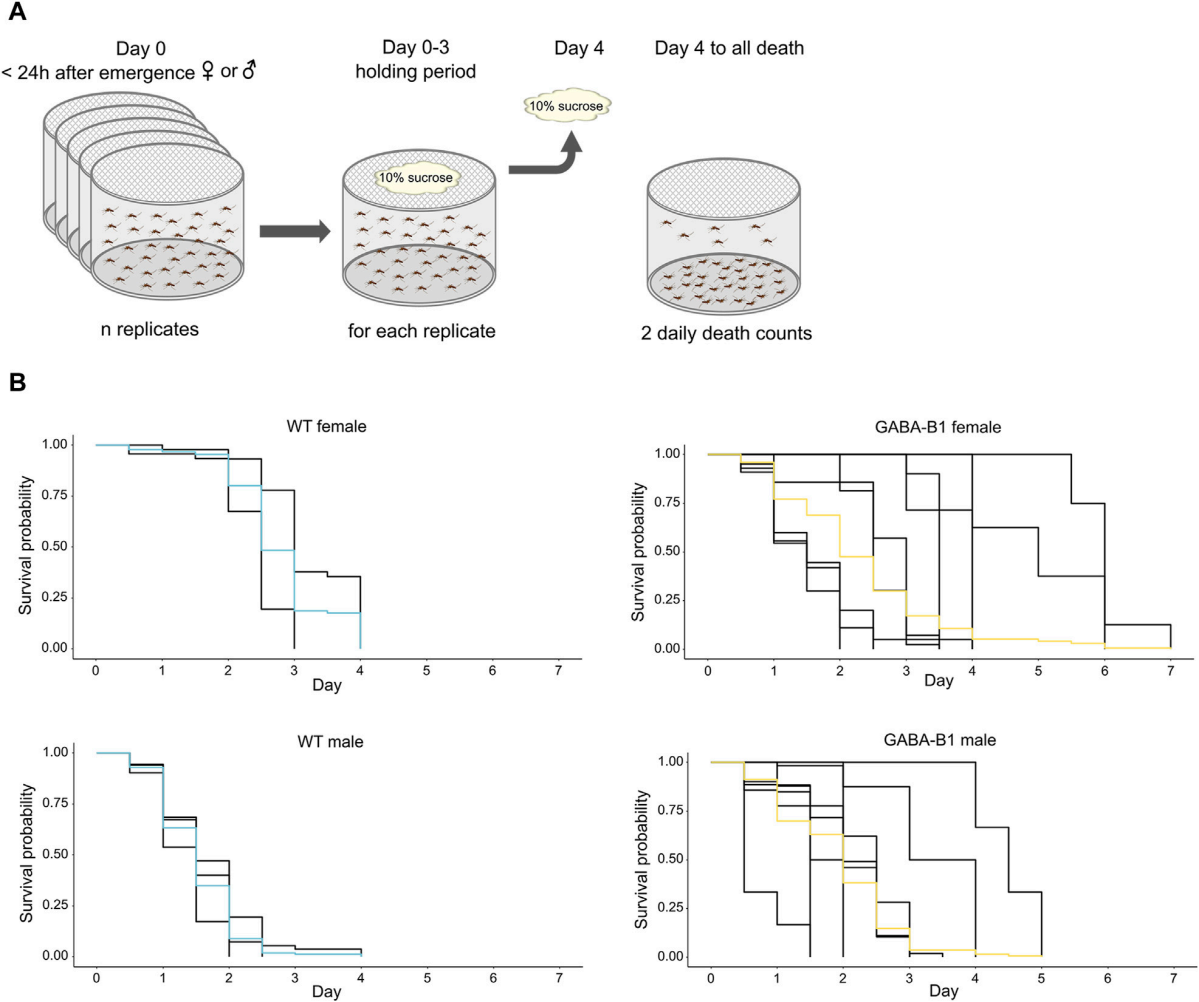
**(A)** Protocol to collect survival data. **(B)** Survival probability over time for each mosquito line after the 10% sucrose is removed. Differences in longevity is compared using Kaplan-Meier analysis. Each black trace corresponds to an individual replicate, and colored curves represent the overall survival probability.

Following an adapted protocol (Reiskind and Lounibos, 2009), within 24 h of emergence, female and male mosquitoes were sorted into different rearing cages (Bugdorm-1, MegaView Science Co., Ltd. Taichung 407008, Taiwan) and stored within a climatic chamber at 27°C, 70%–80% relative humidity, and a photoperiod cycle of 12 h light/12 h dark. Each experiment was replicated three to five times. After the initial 3-day holding period where the mosquitoes were fed on 10% sucrose, and any dead adults were removed, the food was removed. The number of dead mosquitoes was then checked twice a day, both in the morning and evening, until all mosquitoes in the population died. Longevity was defined by the most recent previous time-point at which the individual had been observed alive. Longevity is given in days after the initial 3-day holding period. (Figure 6A).

### 2.5 Olfactory preferences to attractive odors

**FIGURE 7:**
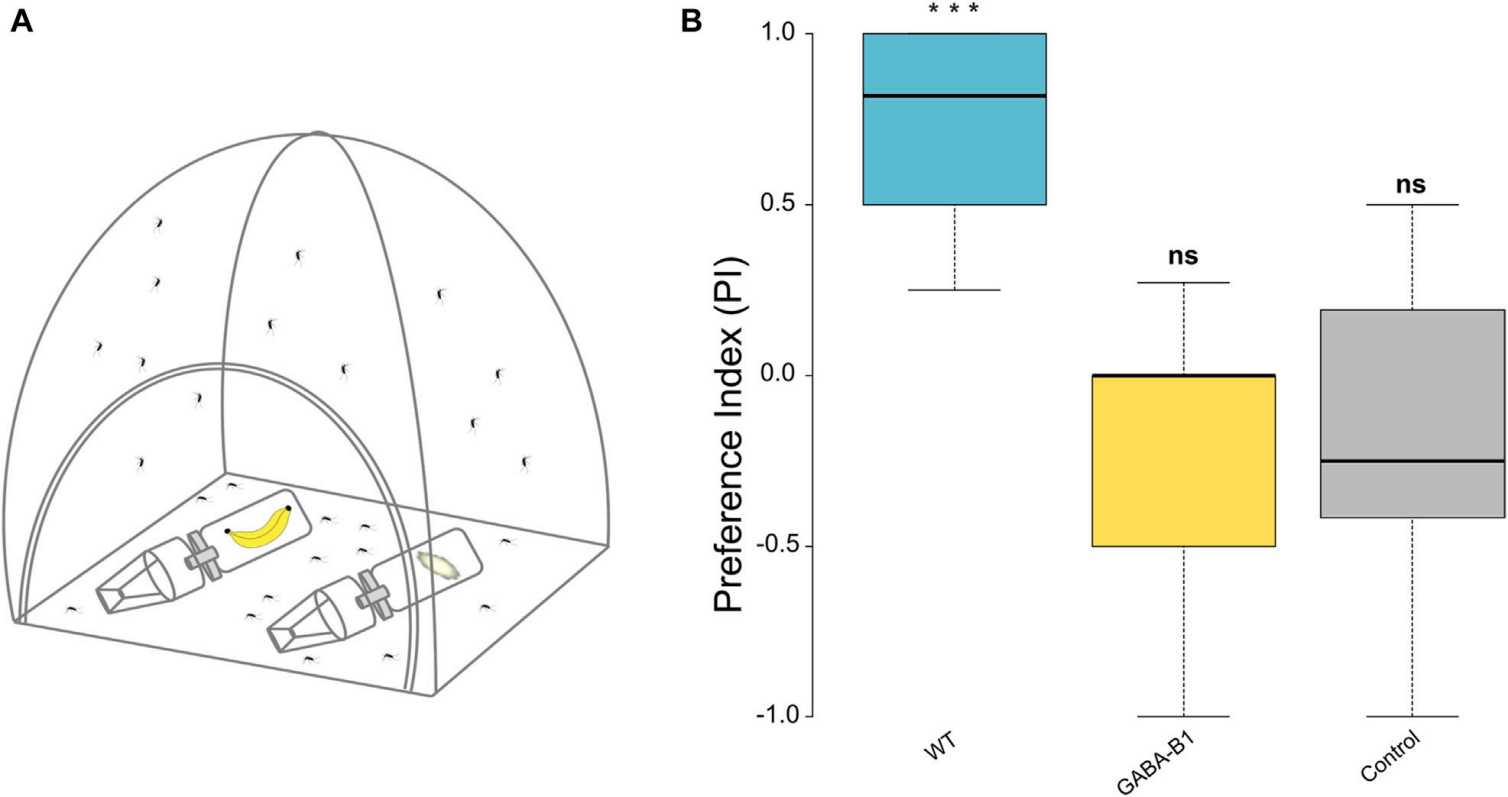
**(A)** Schematic representation of two-choice behavior assay with the experimental and control trap containing whole banana with peel and ball cotton with 10% sucrose, respectively. **(B)** The preference index (PI) was calculated for: wild type (WT), GABA-B1^QF2−ECFP^ (GABA-B1), and control which was performed with the WT line only and 10% sucrose in each trap. A PI of +1 indicates that all motivated mosquitoes chose the attractant container, a PI of 0 indicates that 50% of insects chose the attractant container and 50% the control container, and a PI of −1 indicates that all insects chose the control container. The black line denotes the median value of PI (50th percentile) while the colored boxes contain the 25th to 75th percentiles of PI. The black whiskers mark the 5th and 95th percentiles. The median PI for WT is 0.8 (*n* = 9), for the mutant, it is 0 (*n* = 7) and for the control it is −0.25 (*n* = 15). Asterisks represent statistical significance from zero where *** = *p* < 0.001 and ns for not significant represents non statistical significance from zero.

A custom-made two-choice behavior assay was created to test the response of male and female GABA-B1^QF2−ECFP^ mosquitoes toward a known attractant. To prepare for the experiment, mosquitoes (3–5 day old) were starved overnight, placed inside cages (Bugdorm, 60 cm × 60 cm × 60 cm), and maintained on an inverted light/dark cycle. Our results showed that both males and females were strongly attracted to the trap with the banana odor (exact binomial test, for male *p* = 1.943e-05; for female *p* = 0.0009122) and were not statistically different (Fisher’s exact test, *p* = 0.2564)—we thus included both male and female mosquitoes in our trials. Environmental conditions in the behavior room were 25°C with a relative humidity of 70%–80%. The two-choice behavior assay consisted of a cage (55 × 55 cm) with two smaller traps that had a port in which mosquitoes could enter but not leave. The first container contained the attractant, and the second container contained the control (10% sucrose cotton ball). As an attractant, a banana was chosen as the fruit since it was easy to acquire throughout the year and elicited robust behavioral attraction. Both traps were laid on opposite sides of the cage and the mosquitoes were allowed to choose either the control or experimental trap for 48 h (Figure 7A). Mosquitoes that did not choose either trap were considered as not responsive and not included in the analysis. The placement of the traps was randomized to control for placement biases. The relative humidity difference between each pair of traps was observed to be within 5% of the absolute humidity and, therefore not considered to be attracting the animals. Each experiment was replicated 9 and 7 times for WT and GABA-B1^QF2−ECFP^ lines, respectively. The number of mosquitoes in each trap were counted and included in a Preference Index Assay. The Preference Index (PI) was calculated as (E-C)/(E + C), where E is the number of mosquitoes inside the experimental trap, and C is the number of mosquitoes in the control trap.

### 2.6 Statistical analysis

Analyses were performed in R (version 4.3.0). For the wing size, the dry weight and the egg laying size data, the normal distribution of data was evaluated by the Shapiro-Wilk normality test. Following these results, to compare the wing size between GABA-B1^QF2−ECFP^ and WT line, we used the Student’s t-test while to compare the dry weight and the egg laying size between both lines, we used the Mann-Whitney *U* test. For longevity, Kaplan-Meier analysis of survival with the log-rank test was made on pooled data according to sex and the line and used to do a pairwise comparison between the 2 lines. For the olfactory preference test, a binomial exact test was used to compare the choice of the mosquitoes in the cage between the both traps to a random distribution of 50% of each trap. A Fisher’s exact test was also performed to make a direct comparison between both lines.

## 3 Results

### 3.1 Generating GABA-B1^QF2−ECFP^ transgenic line

To generate a CRISPR/Cas9 mediated HDR knock-in line, *GABA-B1* gene sequence was retrieved from Vectorbase. The *GABA-B1* gene is located on chromosome 2 and spans approximately 473 kb in length [AaegL5_2:233,902,013.234,375,276 (−)], and is composed of a total of 16 protein-coding exons, and two non-coding exons which are located upstream of translational start codon (ATG). The target gRNA sequence is located on protein-coding exon-1 and was selected to undergo HDR and knock-in the DNA cassette downstream of 5′UTR and upstream of the translational start codon. The WT *Ae. aegypti* preblastoderm embryos (*n* = 500) were injected with an equal concentration of Cas9 protein, sgRNA, and knock-in donor plasmid (Figures 1A,B). A total of 337 adults (G_0_) were recovered after the injection and were crossed with WT individuals, and G_1_ eggs were collected. The genomic DNA PCR and Sanger sequencing from G_0_ parents confirmed the integration site for the *GABA-B1* knock-in construct (Supplementary Figure S1). The left-side integration PCR confirmed that the knock-in construct integrated downstream of the 5′UTR sequence and upstream of the translational start codon, whereas the right-side integration PCR confirmed that the knock-in construct integrated 28 base pairs downstream of the translational start codon on coding exon-1 (Supplementary Figure S1). ECFP marker-expressing larvae (Figure 1D) were selected from every generation for inbreeding to establish a homozygous line.

### 3.2 GABA-B1 receptor localization in the mosquito olfactory system

To characterize the GABA-B1 receptor expression within the olfactory region of the *Ae. aegypti* brain, the driver QF2 line was first crossed with the QUAS-mCD8GFP reporter line to allow immunohistochemical localization of the receptor expression. The GFP immunofluorescence revealed low GABA-B1 receptor expression in the calyces and medial and vertical lobes of the MBs compared to outer regions, including the cell bodies. By contrast, the Brp staining clearly showed the different MB structures (Figure 2B). The GABA-B1 receptors were strongly expressed in the AL region. The strongest expression was observed in the lateral and middle cell body clusters surrounding the AL (Figure 2C). These results were consistent and found in male and female mosquitoes, and in both ALs (right and left). Within the AL glomeruli, GABA-B1-expressing neurons showed dendritic bleb-like processes. The GABAergic receptor expression appeared heterogeneous in the AL glomeruli, with higher expression in the outer regions of glomeruli where they receive input from olfactory sensory neurons. In addition, the glomeruli localized in the periphery of the AL show axial projections of neurons expressing GABA-B1 receptors (Figures 2D,E). These neurons do not emanate from the lateral and middle cell clusters but instead originate from a more central or medial area of the brain.

### 3.3 Bioassay

To evaluate the impact of the gene insertion on the fitness of GABA-B1^QF2−ECFP^ transgenic mosquitoes, we performed experiments comparing the size, weight, longevity, and female fecundity with WT mosquitoes.

#### 3.3.1 Transgene insertion correlates with reduced wing size

We examined the effects of transgene insertion on the wing size. For both mutants and WT, the wing size was longer in females than males. In GABA-B1^QF2−ECFP^ females and males, however, the transgene insertion significantly correlated with a reduced wing size compared to WT (for female and male: *p* < 0.001) (Figure 3C).

#### 3.3.2 Transgene insertion correlates with reduced mass

Similar to what was observed for the wing size, males were still smaller than the females in both the WT and GABA-B1^QF2−ECFP^ lines. In addition, the GABA-B1^QF2−ECFP^ line showed significantly lower dry weight in both females and males compared to WT (for female: *p* = 0.004 and for male: *p* = 0.05) (Figure 4B).

#### 3.3.3 Transgene insertion of GABA-B1^QF2−ECFP^ does not impact fecundity

On average, the WT females (*n* = 10) laid 49.6 ± 2.7 eggs while the GABA-B1^QF2−ECFP^ females (*n* = 24) laid 57.9 ± 3.7 eggs. This difference between the WT and the mutant lines was not significantly different (*p* = 0.416) (Figure 5B).

#### 3.3.4 Transgene insertion has an impact on longevity

We compared the longevity of the mutant with WT. For the WT line, the survival probability of males was lower than the survival probability of females, while the survival probability is the same for GABA-B1^QF2−ECFP^ females and males. For the GABA-B1^QF2−ECFP^ females, longevity was significantly shorter compared with the WT females. (WT female vs. GABA-B1^QF2−ECFP^ female: chi2 = 8.10, *p* = 0.004). The median survival probability for GABA-B1^QF2−ECFP^ and WT females was 2 and 2.5 days, respectively. For the GABA-B1^QF2−ECFP^ males, longevity was significantly longer than the WT males, with a median survival probability at 2 and 1.5 days for GABA-B1^QF2−ECFP^ and WT males, respectively (WT male vs. GABA-B1^QF2−ECFP^ male: chi2 = 40.20, *p* < 0.001) (Figure 6B).

### 3.4 Olfactory preferences to attractive odors

The ability of mutant to respond to attractive odors, such as fruit, was evaluated by two-choice behavior assay where the mosquitos are released in a cage and can choose between 2 traps containing either an attractive odor or no odor (control). WT mosquitoes exhibited a strong attraction for the banana (exact binomial test *p* < 0.001). By contrast, the mutant mosquitoes did not show any preference for banana (exact binomial test *p* = 1) and exhibited the same behavior as mosquitoes exposed to the no-odor control (exact binomial test *p* = 0.6208) (Figure 7B). When comparing between treatments, the preference observed between WT and GABA-B1^QF2−ECFP^ groups and the WT and control groups were both significant (Fisher’s exact test, WT vs. GABA-B1^QF2−ECFP^: *p* = 4.518e-05; WT vs. control: *p* = 7.719e-10), while the preference was similar between the GABA-B1^QF2−ECFP^ and the control groups (Fisher’s exact test, GABA-B1^QF2−ECFP^ vs. control: *p* = 1)

## 4 Discussion

We have developed a new transgenic line in one of the main disease vectors, *Ae. aegypti.* Using the CRISPR/Cas9 mediated HDR knock-in approach, the QF2 transcription factor was inserted in the *GABA-B1* receptor gene locus, disrupting the coding sequence of the gene. The GABA-B1^QF2−ECFP^ mutant, expressing the ECFP marker visible in the abdomen at the larval stage, survived and bred in the laboratory over multiple generations without any visible fitness issues, enabling us to establish a homozygous line. This knock-out line phenotype is phenotypically still quite similar to the WT one. The GABA-B1^QF2−ECFP^ individuals show a moderate reduction in size and mass, but not the number of eggs laid. There is a positive correlation between the size and the weight (Packer and Corbet, 1989). Although females of the GABA-B1^QF2−ECFP^ line showed greater variability in survival compared to the WT, male mutant, and WT lines exhibited similar trends in longevity, with the survival probability approaching 0% at approximately 4–5 days. The GABA-B1^QF2−ECFP^ line showed reduced attractiveness to scent emitted from banana fruit odor, which is shown to be attractive to mosquitoes (Paskewitz et al., 2018; Musunzaji et al., 2023).

The reduced attraction to the fruit scent may reflect the importance of GABAergic inhibition in the mosquito olfactory system. The GABA-B1’s heterodimerization with the GABA-B2 subunit is critical for protein structure and function (Galvez et al., 2001; Mezler et al., 2001). In the *Ae. aegypti* mosquito, the antennae have abundant GABA_B_ receptors (Tallon et al., 2019). In *Drosophila*, olfactory receptor neurons (ORNs) express GABA_B_ receptors involved in presynaptic inhibition and reducing the GABA_B_ receptor expression at the presynaptic terminals of ORNs impairs the capability of *Drosophila* flies to locate potential mates (Root et al., 2008). Additionally, the presynaptic inhibition mediated by GABA_B_ receptors offers a mechanism for adjusting olfactory gain. It was demonstrated that the expression of GABA_B_ receptors in ORNs scales the gain of PN responses (Olsen and Wilson, 2008). The work in *Drosophila* is reflected in results from the *Ae. aegypti* mosquito, where GABAergic inhibition in the AL was critical for the processing and the discrimination between attractive and repellent odors. Pharmacological interventions using GABA_B_ receptor antagonists prevented lateral inhibition of specific glomeruli that encoded attractive floral odors (Lahondère et al., 2020).

The GABA-B1^QF2−ECFP^ line was successfully used with the Q-system to drive mCD8GFP expression, allowing characterization of the GABA-B1 receptor expression in the *Ae. aegypti* brain, specifically focusing on loci involved in olfactory processing. The GABA-B1 receptor was not strongly expressed in all brain structures involved in olfactory processing. The receptors showed reduced expression in the MB lobes, although strongly expressed in the MB calyces (Figure 2). The receptors were also strongly expressed in lateral and middle cell body clusters surrounding AL. The peripheral AL glomeruli receive axial projections from neurons expressing GABA receptors localized in central and medial areas of the brain. These results are consistent with earlier studies in *Ae. aegypti*, where the GABA-positive cell bodies are observed in the lateral and ventral clusters around the ALs and in the tracts from the lateral area going into the ALs (Singh et al., 2023). These lateral cell clusters probably correspond to LN clusters as previously found in *Ae. aegypti*. LNs typically project ipsilaterally, and in most of case, their innervation extends to the entire glomerulus. But there is also a small proportion of them which can exhibit a selective innervation to glomeruli. In *Drosophila*, similar to *Ae. aegypti*, many GABA-positive somata are observed in the lateral area of the AL neuropil (Wilson and Laurent, 2005). Among cell bodies bordering the ALs, the dorsolateral cluster contains cell bodies of both PNs and LNs, while anterodorsal cluster contains cell bodies of PNs (Wong et al., 2002; Das et al., 2008; Lai et al., 2008). PNs project from AL to the calyx of MB via the medial antennal lobe tract (mALT), while LNs, showing an ipsilateral projection in the AL, spread their neurites into most glomeruli which contain both presynaptic and postsynaptic connections (Wilson and Laurent, 2005). As mALT neurons have limited presynaptic sites in the AL, LNs are the primary source of GABA signal in this specific *Drosophila* brain region. Moreover, both GABAergic mALT PNs and LNs and non-GABAergic lateral antennal lobe tract (lALT) PNs, projecting from AL to Lateral Horn, express genes of various ionotropic and metabotropic GABA receptor subunits. In addition, metabotropic receptors are expressed in the whole brain with a narrower population of cell bodies around the MB calyx, unlike ionotropic receptors, which are more widely presented in this part of the brain (Okada et al., 2009). In addition, both subunits of GABA_B_ receptors, GABA-B1 and GABA-B2 are co-expressed in similar regions (Okada et al., 2009). GABA receptors are also expressed in the ALs (Enell et al., 2007). The anatomical expression of the GABA_B_ receptor observed in our study also presents similarities with moths. In different moth species, LNs are always situated in the lateral cell clusters and the most multiglomerular LNs in the AL are GABA-positive (Hoskins et al., 1986; Christensen et al., 1993; Anton and Hansson, 1994). In *Bombyx mori*, around 30% of lateral cell body clusters are GABAergic and, like *Ae. aegypti* and *Drosophila*, some of them are LNs that arborize in all AL glomeruli. Axonal PN tracts are also GABA-positive (Hoskins et al., 1986).

The development of the GABA-B1^QF2−ECFP^ mutant line also offers the future ability to characterize and manipulate neuronal functioning. For example, recently developed reporter lines (QUAS-GCaMP7s, QUAS-Crimson, and QUAS-Kir) may allow the characterization of GABA-B1-expressing neurons during odor activation (via QUAS-GCaMP7s), or manipulation of the neuronal activity by crossing the GABA-B1^QF2−ECFP^ line with QUAS-Crimson or QUAS-Kir. The GABA-B1^QF2−ECFP^ mutant line also is a promising tool for examining the neuronal mechanisms of odor representation mediated by GABA inhibition. Indeed, while GABA_A_ receptors shape and synchronize PN activity during the early phase of odor response, GABA_B_ receptors mediate odor-evoked inhibition on the longer time scales (>300 ms) relevant for encoding fluctuating odors that occur in odor plumes (Lei et al., 2002; Wilson and Laurent, 2005). In *Drosophila*, pre-synaptic inhibition of glomeruli encoding attractants (DM2 and DM3) via GABA_B_ receptors has been shown to be important for behavior (Mohamed et al., 2019), although both GABA_B_ and GABA_A_ receptor types are involved in mixture processing. Moreover, the modulation exerted by GABA_B_ receptors is wider, because it involves both pre- and postsynaptic mechanisms (Ozoe, 2013).

Beyond their importance in neural circuit functioning, GABA receptor types are pharmacological targets in insects. The GABA_A_ receptor contains subunits encoded by *Rdl*, which is the site of action for many insecticides. But serious problems of insecticide resistance have been observed, in particular in *Ae. aegypti* populations (Zulfa et al., 2022). As a vector of the pathogens of diseases causing 96 million new cases and around 40,000 deaths every year in the case of dengue alone (World Health Organization, 2020), there is an urgent need to investigate other insecticide targets. The GABA-B1 receptor is a promising target as it is involved in vital physiological mechanisms. In *Drosophila,* the developmental role of GABA-B1 receptors was demonstrated using an injectable RNAi method. Initially designed for selective gene suppression in adult flies, the method was adapted for embryos. Injections of double-stranded RNA targeting GABA-B1 or a GABA_B_ antagonist CGP54626 resulted in lethality by the third larval stage. The affected larvae were smaller, with compressed and folded tracheae, suggesting that GABA_B_ receptor antagonism disrupts the molting process during development (Dzitoyeva et al., 2005). GABA_B_ receptors also play a role in regulating circadian rhythms through the clock neurons and neuronal circuits of the circadian system. It was demonstrated that the master clock neurons, s-LN(v)s, utilize GABA as a slow inhibitory neurotransmitter that can be blocked by a GABA_B_ antagonist (Hamasaka et al., 2005). GABA_B_ receptors have been identified as pharmacological targets (Bowery, 2006) and could bring new solutions to manage vector populations.

In conclusion, the development of the GABA-B1^QF2−ECFP^ mutant line in *Ae. aegypti*, achieved through CRISPR/Cas9-mediated HDR knock-in, offers a valuable new neurogenetic tool for investigating the role of the GABAergic systems within the central olfactory system and will help us to investigate its role in the processing of attractive odors. The observed changes in attractiveness to fruit scents emphasize the importance of the GABA-B1 receptor in mosquito olfaction. Consequently, GABA-B1 receptors can be considered as potential pharmacological targets that present promising prospects for addressing insecticide resistance and developing novel strategies for vector population control.

## Data Availability

The original contributions presented in the study are included in the article/Supplementary Material, further inquiries can be directed to the corresponding author.

## Supplementary material

SUPPLEMENTARY TABLE S1 List of oligos used in this study.

SUPPLEMENTARY FIGURE S1 Genotyping of GABA-B1^QF2−ECFP^ line. **(A and B)** The schematic representation of the *GABA-B1* gene structure and homology-directed repair (HDR) knock-in construct. The black arrow with F1-R1 and F2-R2 represents the primers used for genotyping PCR to determine the left and right-side integration of knock-in. **(C)** PCR products obtained from genotyping PCR were analyzed on 1% agarose gel and their nucleotide sequences were confirmed by Sanger sequencing. Left and right-side integration PCR performed using the GABA-B1^QF2−ECFP^ line genomic DNA template, whereas negative control PCR was performed using wild type genomic DNA template or no template. **(D)** The nucleotide sequence alignment of the left and right-side integration PCR confirmed the precise genomic insertion of the knock-in construct. The knock-in construct (top nucleotide sequences) is used as a reference sequence to align with the left and right site integration PCR sequence (bottom nucleotide sequences and chromatograms).

## References

[B1] Anton S. Hansson B. S. (1994). Central processing of sex pheromone, host odour, and oviposition deterrent information by interneurons in the antennal lobe of female Spodoptera littoralis (Lepidoptera: noctuidae). J. Comp. Neurology 350, 199–214. 10.1002/cne.903500205 7884038

[B2] Bowery N. G. (2006). GABAB receptor: a site of therapeutic benefit. Curr. Opin. Pharmacol. 6, 37–43. 10.1016/j.coph.2005.10.002 16361115

[B3] Brotz T. M. Gundelfinger E. D. Borst A. (2001). Cholinergic and GABAergic pathways in fly motion vision. BMC Neurosci. 2, 1. 10.1186/1471-2202-2-1 11242563 PMC29101

[B4] Bui M.A.-Li M.A.-Raban R. R. A.-Liu N.A.-Akbari O. S. A.- (2020). Embryo microinjection techniques for efficient site-specific mutagenesis in *Culex quinquefasciatus* . JoVE, e61375. 10.3791/61375 32510506

[B5] Casida J. E. Durkin K. A. (2015). Novel GABA receptor pesticide targets. Pesticide Biochem. Physiology 121, 22–30. 10.1016/j.pestbp.2014.11.006 26047108

[B6] CHOPCHOP (2024). CHOPCHOP. Available at: https://chopchop.cbu.uib.no/(Accessed January 23, 2024).

[B7] Christensen T. A. Waldrop B. R. Harrow I. D. Hildebrand J. G. (1993). Local interneurons and information processing in the olfactory glomeruli of the moth Manduca sexta. J. Comp. Physiology A 173, 385–399. 10.1007/BF00193512 8254565

[B8] Das A. Sen S. Lichtneckert R. Okada R. Ito K. Rodrigues V. (2008). Drosophila olfactory local interneurons and projection neurons derive from a common neuroblast lineage specified by the empty spiracles gene. Neural Dev. 3, 33. 10.1186/1749-8104-3-33 19055770 PMC2647541

[B9] Database (2024). Insect brain Database. Available at: https://insectbraindb.org/app/(Accessed January 23, 2024).

[B10] Davis R. L. (1993). Mushroom bodies and drosophila learning. Neuron 11, 1–14. 10.1016/0896-6273(93)90266-T 8338661

[B63] Dupuis J. P. Bazelot M. Barbara G. S. Paute S. Gauthier M. Raymond-Delpech V. (2010). Homomeric RDL and heteromeric RDL/LCCH3 GABA receptors in the honeybee antennal lobes: Two candidates for inhibitory transmission in olfactory processing. J. of Neurophys. 103, 458–468. 10.1152/jn.00798.2009 19906878

[B11] Dzitoyeva S. Gutnov A. Imbesi M. Dimitrijevic N. Manev H. (2005). Developmental role of GABAB(1) receptors in Drosophila. Dev. Brain Res. 158, 111–114. 10.1016/j.devbrainres.2005.06.005 16054235

[B12] Enell L. Hamasaka Y. Kolodziejczyk A. Nässel D. R. (2007). gamma-Aminobutyric acid (GABA) signaling components in Drosophila: immunocytochemical localization of GABA(B) receptors in relation to the GABA(A) receptor subunit RDL and a vesicular GABA transporter. J. Comp. Neurology 505, 18–31. 10.1002/cne.21472 17729251

[B13] Ffrench-Constant R. H. Mortlock D. P. Shaffer C. D. MacIntyre R. J. Roush R. T. (1991). Molecular cloning and transformation of cyclodiene resistance in Drosophila: an invertebrate gamma-aminobutyric acid subtype A receptor locus. Proc. Natl. Acad. Sci. 88, 7209–7213. 10.1073/pnas.88.16.7209 1651498 PMC52263

[B14] Freifeld L. Clark D. A. Schnitzer M. J. Horowitz M. A. Clandinin T. R. (2013). GABAergic lateral interactions tune the early stages of visual processing in Drosophila. Neuron 78, 1075–1089. 10.1016/j.neuron.2013.04.024 23791198 PMC3694283

[B15] Galvez T. Duthey B. Kniazeff J. Blahos J. Rovelli G. Bettler B. (2001). Allosteric interactions between GB1 and GB2 subunits are required for optimal GABAB receptor function. EMBO J. 20, 2152–2159. 10.1093/emboj/20.9.2152 11331581 PMC125244

[B16] Gibson D. G. Young L. Chuang R.-Y. Venter J. C. Hutchison C. A. Smith H. O. (2009). Enzymatic assembly of DNA molecules up to several hundred kilobases. Nat. Methods 6, 343–345. 10.1038/nmeth.1318 19363495

[B17] Hamasaka Y. Wegener C. Nässel D. R. (2005). GABA modulates Drosophila circadian clock neurons via GABAB receptors and decreases in calcium. J. Neurobiol. 65, 225–240. 10.1002/neu.20184 16118795

[B18] Harvey R. J. Schmitt B. Hermans-Borgmeyer I. Gundelfinger E. D. Betz H. Darlison M. G. (1994). Sequence of a Drosophila ligand-gated ion-channel polypeptide with an unusual amino-terminal extracellular domain. J. Neurochem. 62, 2480–2483. 10.1046/j.1471-4159.1994.62062480.x 8189252

[B19] Heisenberg M. Borst A. Wagner S. Byers D. (1985). Drosophila mushroom body mutants are deficient in olfactory learning. J. Neurogenetics 2, 1–30. 10.3109/01677068509100140 4020527

[B20] Henderson J. E. Soderlund D. M. Knipple D. C. (1993). Characterization of a putative gamma-aminobutyric acid (GABA) receptor beta subunit gene from *Drosophila melanogaster* . Biochem. Biophysical Res. Commun. 193, 474–482. 10.1006/bbrc.1993.1648 7685594

[B21] Hofbauer A. Ebel T. Waltenspiel B. Oswald P. Chen Y. Halder P. (2009). The wuerzburg hybridoma library against Drosophila brain. J. Neurogenetics 23, 78–91. 10.1080/01677060802471627 19132598

[B22] Homberg U. (2002). Neurotransmitters and neuropeptides in the brain of the locust. Microsc. Res. Tech. 56, 189–209. 10.1002/jemt.10024 11810722

[B23] Hosie A. Sattelle D. Aronstein K. ffrench-Constant R. (1997). Molecular biology of insect neuronal GABA receptors. Trends Neurosci. 20, 578–583. 10.1016/S0166-2236(97)01127-2 9416671

[B24] Hoskins S. G. Homberg U. Kingan T. G. Christensen T. A. Hildebrand J. G. (1986). Immunocytochemistry of GABA in the antennal lobes of the sphinx moth Manduca sexta. Cell. Tissue Res. 244, 243–252. 10.1007/BF00219199 3521878

[B25] Hsu C. T. Bhandawat V. (2016). Organization of descending neurons in *Drosophila melanogaster* . Sci. Rep. 6, 20259. 10.1038/srep20259 26837716 PMC4738306

[B26] Lahondère C. Vinauger C. Okubo R. P. Wolff G. H. Chan J. K. Akbari O. S. (2020). The olfactory basis of orchid pollination by mosquitoes. Proc. Natl. Acad. Sci. 117, 708–716. 10.1073/pnas.1910589117 31871198 PMC6955360

[B27] Lai S.-L. Awasaki T. Ito K. Lee T. (2008). Clonal analysis of Drosophila antennal lobe neurons: diverse neuronal architectures in the lateral neuroblast lineage. Development 135, 2883–2893. 10.1242/dev.024380 18653555

[B28] Leal S. M. Neckameyer W. S. (2002). Pharmacological evidence for GABAergic regulation of specific behaviors in *Drosophila melanogaster* . J. Neurobiol. 50, 245–261. 10.1002/neu.10030 11810639

[B29] Lei H. Christensen T. A. Hildebrand J. G. (2002). Local inhibition modulates odor-evoked synchronization of glomerulus-specific output neurons. Nat. Neurosci. 5, 557–565. 10.1038/nn0602-859 12006983

[B30] Li M. Yang T. Bui M. Gamez S. Wise T. Kandul N. P. (2021). Suppressing mosquito populations with precision guided sterile males. Nat. Commun. 12, 5374. 10.1038/s41467-021-25421-w 34508072 PMC8433431

[B31] Li M. Yang T. Kandul N. P. Bui M. Gamez S. Raban R. (2020). Development of a confinable gene drive system in the human disease vector *Aedes aegypti* . eLife 9, e51701. 10.7554/eLife.51701 31960794 PMC6974361

[B32] Liou N.-F. Lin S.-H. Chen Y.-J. Tsai K.-T. Yang C.-J. Lin T.-Y. (2018). Diverse populations of local interneurons integrate into the Drosophila adult olfactory circuit. Nat. Commun. 9, 2232. 10.1038/s41467-018-04675-x 29884811 PMC5993751

[B33] Liu X. Davis R. L. (2009). The GABAergic anterior paired lateral neuron suppresses and is suppressed by olfactory learning. Nat. Neurosci. 12, 53–59. 10.1038/nn.2235 19043409 PMC2680707

[B34] Loh Y. M. Su M. P. Ellis D. A. Andrés M. (2023). The auditory efferent system in mosquitoes. Front. Cell. Dev. Biol. 11, 1123738. 10.3389/fcell.2023.1123738 36923250 PMC10009176

[B35] Mezler M. Müller T. Raming K. (2001). Cloning and functional expression of GABAB receptors from Drosophila. Eur. J. Neurosci. 13, 477–486. 10.1046/j.1460-9568.2001.01410.x 11168554

[B36] Mohamed A. A. M. Retzke T. Das Chakraborty S. Fabian B. Hansson B. S. Knaden M. (2019). Odor mixtures of opposing valence unveil inter-glomerular crosstalk in the Drosophila antennal lobe. Nat. Commun. 10, 1201. 10.1038/s41467-019-09069-1 30867415 PMC6416470

[B37] Musunzaji P. S. Ndenga B. A. Mzee S. Abubakar L. U. Kitron U. D. Labeaud A. D. (2023). Oviposition preferences of *Aedes aegypti* in msambweni, kwale county, Kenya. J. Am. Mosquito Control Assoc. 39, 85–95. 10.2987/22-7103 PMC10885850 37270926

[B38] Okada R. Awasaki T. Ito K. (2009). Gamma-aminobutyric acid (GABA)-mediated neural connections in the Drosophila antennal lobe. J. Comp. neurology 514, 74–91. 10.1002/cne.21971 19260068

[B39] Olsen S. R. Wilson R. I. (2008). Lateral presynaptic inhibition mediates gain control in an olfactory circuit. Nature 452, 956–960. 10.1038/nature06864 18344978 PMC2824883

[B40] Ozoe Y. (2013). “Chapter four - γ-aminobutyrate- and glutamate-gated chloride channels as targets of insecticides,” in Advances in insect Physiology. Editor Cohen E. (Germany: Academic Press), 211–286. 10.1016/B978-0-12-394389-7.00004-1

[B41] Ozoe Y. (2021). Ion channels and G protein-coupled receptors as targets for invertebrate pest control: from past challenges to practical insecticides. Biosci. Biotechnol. Biochem. 85, 1563–1571. 10.1093/bbb/zbab089 33988673

[B42] Packer M. J. Corbet P. S. (1989). Size variation and reproductive success of female Aedes punctor (Diptera: Culicidae). Ecol. Entomol. 14, 297–309. 10.1111/j.1365-2311.1989.tb00960.x

[B43] Paskewitz S. Irwin P. Konwinski N. Larson S. (2018). Impact of consumption of bananas on attraction of *Anopheles stephensi* to humans. Insects 9, 129. 10.3390/insects9040129 30274200 PMC6315685

[B44] Pelizza S. A. Scorsetti A. C. Tranchida M. C. (2013). The sublethal effects of the entomopathic fungus Leptolegnia chapmanii on some biological parameters of the dengue vector *Aedes aegypti* . J. Insect Sci. 13, 22. 10.1673/031.013.2201 23901823 PMC3735114

[B45] Primordium (2023). Primordium labs. Available at: https://www.primordiumlabs.com/ (Accessed January 23, 2024).

[B46] Raghu S. V. Claussen J. Borst A. (2013). Neurons with GABAergic phenotype in the visual system of Drosophila. J. Comp. Neurology 521, 252–265. 10.1002/cne.23208 22886821

[B47] Reiskind M. H. Lounibos L. P. (2009). Effects of intraspecific larval competition on adult longevity in the mosquitoes *Aedes aegypti* and *Aedes albopictus* . Med. Veterinary Entomology 23, 62–68. 10.1111/j.1365-2915.2008.00782.x PMC2651082 19239615

[B48] Riabinina O. Task D. Marr E. Lin C.-C. Alford R. O’Brochta D. A. (2016). Organization of olfactory centres in the malaria mosquito *Anopheles gambiae* . Nat. Commun. 7, 13010. 10.1038/ncomms13010 27694947 PMC5063964

[B49] Rogers S. M. Matheson T. Sasaki K. Kendrick K. Simpson S. J. Burrows M. (2004). Substantial changes in central nervous system neurotransmitters and neuromodulators accompany phase change in the locust. J. Exp. Biol. 207, 3603–3617. 10.1242/jeb.01183 15339956

[B50] Root C. M. Masuyama K. Green D. S. Enell L. E. Nässel D. R. Lee C.-H. (2008). A presynaptic gain control mechanism fine-tunes olfactory behavior. Neuron 59, 311–321. 10.1016/j.neuron.2008.07.003 18667158 PMC2539065

[B51] Shankar S. McMeniman C. J. (2020). An updated antennal lobe atlas for the yellow fever mosquito *Aedes aegypti* . PLOS Neglected Trop. Dis. 14, e0008729. 10.1371/journal.pntd.0008729 PMC7575095 33079925

[B52] Singh P. Goyal S. Gupta S. Garg S. Tiwari A. Rajput V. (2023). Combinatorial encoding of odors in the mosquito antennal lobe. Nat. Commun. 14, 3539. 10.1038/s41467-023-39303-w 37322224 PMC10272161

[B53] Stopfer M. Bhagavan S. Smith B. Laurent G. (1997). Impaired odour discrimination on desynchronization of odour-encoding neural assemblies. Nature 390, 70–74. 10.1038/36335 9363891

[B54] Tallon A. K. Hill S. R. Ignell R. (2019). Sex and age modulate antennal chemosensory-related genes linked to the onset of host seeking in the yellow-fever mosquito, *Aedes aegypti* . Sci. Rep. 9, 43. 10.1038/s41598-018-36550-6 30631085 PMC6328577

[B55] Tastekin I. Khandelwal A. Tadres D. Fessner N. D. Truman J. W. Zlatic M. (2018). Sensorimotor pathway controlling stopping behavior during chemotaxis in the *Drosophila melanogaster* larva. eLife 7, e38740. 10.7554/eLife.38740 30465650 PMC6264072

[B56] VectorBase (2022). VectorBase. Available at: https://vectorbase.org/vectorbase/app (Accessed January 23, 2024).

[B57] Waldrop B. Christensen T. Hildebrand J. (1987). GABA-mediated synaptic inhibition of projection neurons in the antennal lobes of the sphinx moth, Manduca sexta. J. Comp. physiology. A, Sens. neural, Behav. physiology 161, 23–32. 10.1007/bf00609452 3039128

[B58] Wilson R. I. Laurent G. (2005). Role of GABAergic inhibition in shaping odor-evoked spatiotemporal patterns in the Drosophila antennal lobe. J. Neurosci. 25, 9069–9079. 10.1523/JNEUROSCI.2070-05.2005 16207866 PMC6725763

[B59] Wong A. M. Wang J. W. Axel R. (2002). Spatial representation of the glomerular map in the Drosophila protocerebrum. Cell. 109, 229–241. 10.1016/S0092-8674(02)00707-9 12007409

[B60] World Health Organization (2020). Vector-borne diseases. Available at: https://www.who.int/news-room/factsheets/.

[B61] Yasuyama K. Meinertzhagen I. A. Schürmann F.-W. (2002). Synaptic organization of the mushroom body calyx in *Drosophila melanogaster* . J. Comp. Neurology 445, 211–226. 10.1002/cne.10155 11920702

[B62] Zulfa R. Lo W.-C. Cheng P.-C. Martini M. Chuang T.-W. (2022). Updating the insecticide resistance status of *Aedes aegypti* and *Aedes albopictus* in asia: a systematic review and meta-analysis. Trop. Med. Infect. Dis. 7, 306. 10.3390/tropicalmed7100306 36288047 PMC9607256

