# Supplementary figures and images for: Transgenic line for characterizing GABA-receptor expression to study the neural basis of olfaction in the yellow-fever mosquito
Source: Front Physiol. 2024 Mar 28;15:1381164. doi: 10.3389/fphys.2024.1381164 (PMC11008680; doi:10.3389/fphys.2024.1381164)

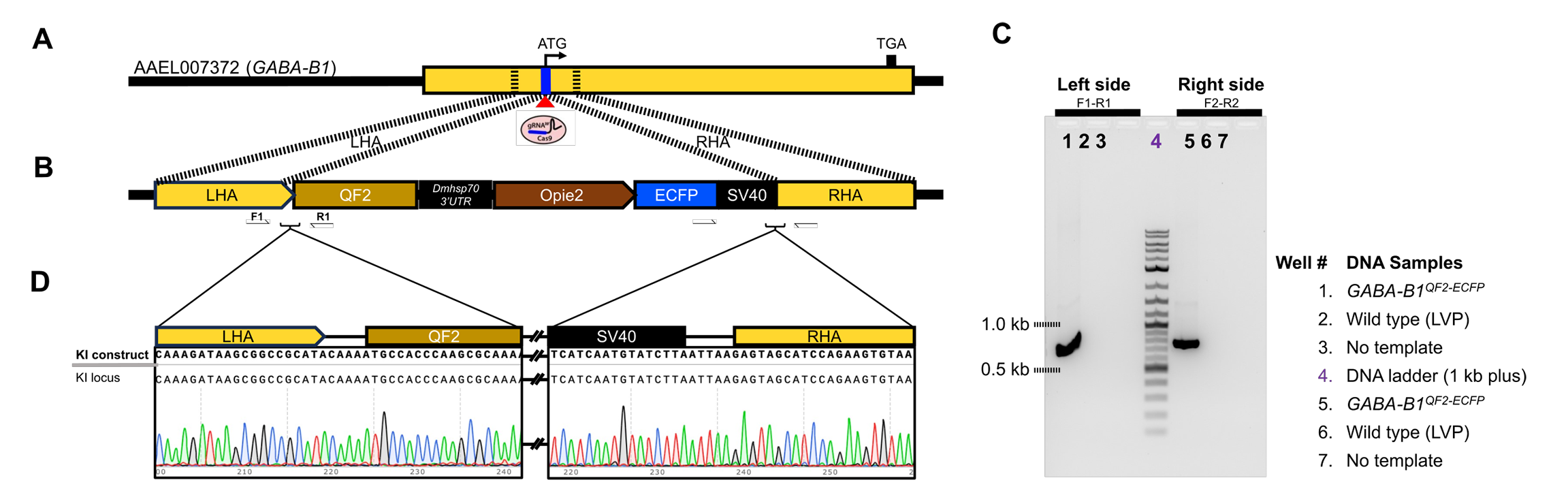

Supplement: Supplementary file 1 [file Image1.tif]
